# Standardizing outcomes in food allergy research: aligning clinical trials with patient priorities

**DOI:** 10.1097/ACI.0000000000001074

**Published:** 2025-04-21

**Authors:** Daniel Munblit, Christian Apfelbacher, Anastasia Demidova, Audrey DunnGalvin, Robert J. Boyle

**Affiliations:** a Care for Long Term Conditions Division, Florence Nightingale Faculty of Nursing, Midwifery and Palliative Care, King's College London, London, UK; b Department of Paediatrics and Paediatric Infectious Diseases, Institute of Child's Health, Sechenov First Moscow State Medical University (Sechenov University), Moscow, Russia; c Institute of Social Medicine and Health Systems Research, Otto von Guericke University, Medical Faculty, Magdeburg, Germany; d Independent Researcher, Cyprus; e School of Applied Psychology, University College Cork, Cork, Ireland; f National Heart and Lung Institute, Imperial College London, London, UK

**Keywords:** core outcome set, desensitization, food allergy, immunotherapy, outcome heterogeneity, patient-centred outcomes, quality of life, sustained unresponsiveness

## Abstract

**Purpose of review:**

This review explores the clinical outcomes used in immunoglobulin E (IgE)-mediated food allergy (FA) intervention studies, emphasizing unmet need for patient-centred outcomes. Standardizing outcome measurement is critical as research into FA treatments, particularly food immunotherapy, expands. Here we discuss how outcomes should reflect the multidimensional impact of FA on people's lives.

**Recent findings:**

Current evidence reveals a discrepancy between clinical trial outcomes and those most valued by patients and carers. While trials often prioritize changes in reactivity thresholds or immunological markers, patients and carers emphasize need in reducing severe reactions, improving quality of life, and enhancing confidence in disease management. This disparity highlights importance of harmonization efforts to guide FA research.

The Core Outcome Measures for Food Allergy (COMFA) initiative recently identified two core outcomes – ‘allergic symptoms’ and ‘quality of life’ – through an international consensus process involving patients, caregivers, clinicians, and researchers. Outcomes like ‘desensitization’ and ‘remission/sustained unresponsiveness’ were considered important but were not seen as the most critical.

**Summary:**

Developing and implementing a COS for FA intervention studies is essential to align research with patient priorities, ensuring meaningful improvements in routine clinical care. Standardized outcome measurement will generate robust evidence, inform clinical practice, and empower patients and caregivers in decision-making about FA management.

## INTRODUCTION

Food allergy (FA) represents a significant global public health concern, with prevalence estimates varying between 0.8% and 13%, depending on whether the data is self-reported or confirmed via oral food challenge [1]. FA is associated with a substantial economic [2], social, and emotional burden [3] on those affected and their families and friends, often leading to reduced quality of life (QoL). Traditional management strategies, focused on allergen avoidance have been suggested to be suboptimal, as accidental exposures remain common and can occasionally result in severe reactions [4,5].

Recent years have seen a rise in interest in novel FA interventions, with a strong focus on allergen immunotherapy (AIT) and biologic therapies, which both aim to suppress the immune response to food allergens. Different AIT modalities, such as oral immunotherapy (OIT) or epicutaneous immunotherapy (EPIT), have demonstrated ability to induce desensitization in individuals with FA, increasing threshold at which individuals react to allergens [6^▪^]. However, while AIT often successfully achieves desensitization, its ability to lead to a sustained unresponsiveness (SU) – a state of clinical ‘nonreactivity’ to an allergen after completing AIT is uncertain. Achievement of SU is understandably a more desirable outcome for patients and their families [7], as it suggests long-term tolerance to the allergen without the need for continued treatment.

While the key focus of current research is the development of safe and effective treatments, there is a substantial variability in the efficacy outcomes reported across the trials, leading to ongoing debates about which outcome measures are most important and how they should be standardized [8^▪^,^9^]. This heterogeneity in outcome reporting brings challenges for comparing efficacy and safety results across studies and evaluating the overall efficacy of different treatments in meta-analyses which in turn impacts guideline development processes.

In order to address outcome heterogeneity, the Core Outcome Set (COS) concept has been proposed as a potential solution. A COS is an agreed standardized set of outcomes that should be measured and reported, as a minimum, in specific areas of health or healthcare [10]. Developed through consensus involving key stakeholders, including patients, healthcare professionals, and researchers, a COS – if widely adopted - ensures that outcomes of utmost importance are consistently evaluated across studies [11]. Recently, the Core Outcome Measures for Food Allergy (COMFA) initiative developed a Core Outcome Set (COS) for FA [12^▪▪^]. Through an international, multidisciplinary consensus process, COMFA has identified ’allergic symptoms’ and QoL’ as critical outcomes to be assessed in all clinical trials and observational studies of interventions.

This review discusses the current landscape of outcomes used in IgE-mediated FA intervention studies, highlighting the need to prioritize patient-relevant outcomes. 

**Box 1:**
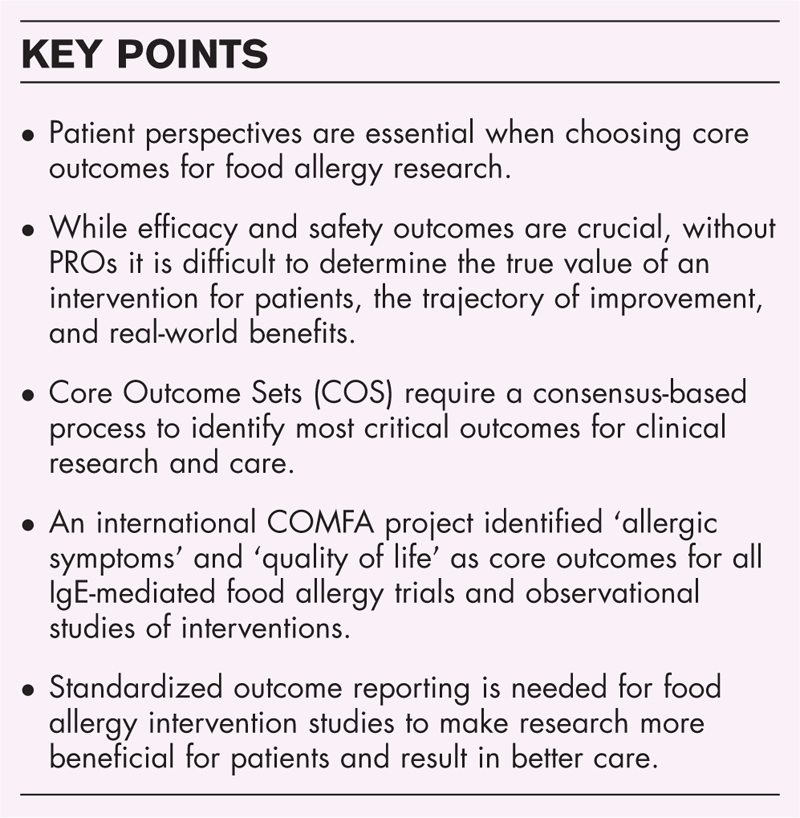
no caption available

## THE CURRENT OUTCOME LANDSCAPE IN FOOD ALLERGY TRIALS

Trials of FA interventions, particularly food immunotherapy, have traditionally focused on measuring biomarkers and surrogate outcomes, which remains relevant for the recently conducted trials (Table 1). These include measures such as the threshold of reactivity during a supervised, graded oral food challenge, the classification of food challenge reaction outcomes, and the proportion of participants experiencing treatment-related adverse events [9]. Desensitization and SU remain two key efficacy outcomes commonly evaluated in FA trials [13^▪▪^].

**Table 1:** Recent key clinical trials on food allergy treatments and their outcomes

Author, year	Trial name	Intervention	Primary efficacy outcome	Secondary outcomes assessed	Definition of desensitization	Definition of sustained unresponsiveness	Quality of Life
OIT							
Vickery *et al.* 2018 [44]	PALISADE	Peanut	67.2% of participants in the active group tolerated ≥600 mg of peanut protein vs. 4.0% in the placebo group (difference: 63.2%; 95% CI: 53.0–73.3; *P* < 0.001).	- Proportion tolerating 300 mg and 1000 mg of peanut protein during the exit OFC - Maximum severity of symptoms during the exit OFC - Rescue epinephrine use	Ability of participants to tolerate a cumulative dose of 600 mg or more of peanut protein during the exit DBPCFC, without experiencing dose-limiting symptoms, after completing the treatment regimen	Not assessed	Not reported^a^
Chinthrajah *et al.* 2019 [45]	POISED	Peanut	35% of participants in the peanut-0 group tolerated 4000 mg of peanut protein at weeks 104 and 117 vs. 4% in the placebo group (OR: 12.7; 95% CI: 1.8–554.8; P = 0.0024).	- Proportion of participants passing DBPCFC at weeks 104, 130, 143, and 156 - Tolerability and severity of symptoms during build-up and maintenance phases - Success rates of those maintained on 300 mg daily peanut protein vs. complete discontinuation - Cumulative tolerated dose at each timepoint and associated clinical reactivity	Ability to consume 4000 mg peanut protein without experiencing objective symptoms, achieved during therapy while actively consuming peanut protein daily	Ability to consume 4000 mg peanut protein without experiencing objective symptoms 3–12 months after discontinuing OIT	Not reported
Hourihane *et al.* 2020 [46]	ARTEMIS	Peanut	58% of participants in the active group tolerated 1000 mg (cumulative 2043 mg) of peanut protein vs. 2% in the placebo group (difference: 56%; *P* < 0.001)	- Proportion tolerating 600 mg and 300 mg doses of peanut protein at the exit OFC - Severity of symptoms during OFC - Food allergy-related quality of life (measured by FAQLQ and FAIM)	Temporary ability to tolerate 1000 mg or more of peanut protein without dose-limiting symptoms while on therapy	Not assessed	Reported (FAQLQ and FAIM)
Maeda *et al.* 2021 [47]	ORIMA	Whole cow's milk	50% of participants in the intervention group tolerated 100 mL of cow's milk vs. 0% in the control group (*P* < 0.01) at the 12-month DBPCFC	- Increase in cow's milk tolerance threshold - Percentage change in threshold - Changes in cow's milk-specific IgE, casein-specific IgE, and β-lactoglobulin-specific IgE levels - Skin prick test results - Eosinophil counts - Adverse events and adrenaline use - Long-term follow-up (2 years poststudy)	Ability to tolerate 100 mL of cow's milk without reactions or only mild symptoms during active treatment, as confirmed by DBPCFC at 12 months	Not specifically defined in trial. Long-term follow-up for persistence of tolerance has been assessed.	Not reported
Dantzer *et al.* 2022 [48]	Not reported	Baked milk	73% of participants in the baked milk OIT group tolerated 4044 mg of baked milk protein vs. 0% in the placebo group	- Increase in maximum tolerated dose of baked milk protein from baseline to 12 months - Immunological changes (cow's milk-sIgG4 and casein-specific IgE levels) - Quality of life	Ability to tolerate 4044 mg of baked milk protein (equivalent to a cumulative dose of baked milk) without experiencing dose-limiting symptoms during the DBPCFC at 12 months	Not assessed. Longer-term unblinded follow-up will evaluate tolerance to baked and unheated milk up to 8 g.	Reported (FAQLQ (Parent Form, Child Form, Teenager Form, and Adult Form))
EPIT							
Sampson *et al.* 2017 [49]	VIPES	Peanut patch	50% of participants in the active group achieved a ≥10-fold increase in ED or an ED ≥1000 mg vs. 25% in the placebo group (difference: 25.0%; 95% CI: 7.7–42.3; *P* = 0.01)	- Increased cumulative reactive dose in responders in the 250 μg group compared to placebo - Immunological changes (peanut-specific IgG4 and peanut-specific IgE) - Safety outcomes focused on local skin reactions and systemic adverse events	Ability to achieve a ≥10-fold increase in ED for participants with a baseline ED ≤300 mg, or to tolerate an ED ≥1000 mg for participants with a baseline ED >300 mg, during the 12-month DBPCFC	Not assessed	Not reported^a^
Fleischer *et al.* 2019 [50]	PEPITES	Peanut patch	67.2% of participants in the AR101 group tolerated ≥600 mg of peanut protein vs. 4.0% in the placebo group (difference: 63.2%; 95% CI: 53.0–73.3; *P* < 0.001)	- Responder rate differences between eliciting dose subgroups - Cumulative reactive dose differences between treatment groups - Immunological changes in peanut-specific IgE and IgG4 levels	Ability to tolerate a higher eliciting dose of peanut protein (≥300 mg or ≥1000 mg) at the 12-month DBPCFC	Not assessed	Not reported^a^
Greenhawt *et al.* 2023 [16^▪▪^]	EPITOPE	Peanut patch	67.0% of participants in the intervention group met the responder endpoint compared to 33.5% in the placebo group (difference: 33.4 percentage points, 95% CI, 22.4–44.5; *P* < 0.001)	- Increase in CRD and ED from baseline to month 12 - Median change in CRD - Median change in ED - Immunological changes: (peanut-specific IgG4 and peanut-specific IgE) - Symptom severity during food challenges	Ability to tolerate at least 1000 mg of peanut protein (for baseline ED >10 mg) or a ≥300 mg ED (for baseline ED ≤10 mg) after 12 months of therapy, as measured by DBPCFC	Not assessed	Not reported^a^
Omalizumab							
Wood *et al.* 2024 [18^▪▪^]	OUtMATCH	Omalizumab	67% of participants in the omalizumab group achieved the outcome compared to 7% in the placebo group (difference: 60 percentage points; 95% CI: 47–70; *P* < 0.001)	- Consumption of at least 1000 mg of cashew, milk, or egg protein without dose-limiting symptoms - Quality of life - Safety outcomes including injection-site reactions	Ability to consume a single dose of 600 mg of peanut protein without dose-limiting symptoms at 16 weeks	Not assessed	Reported FAQLQ (Total Score and Total Parent Score)

a Included in the study protocol as a secondary outcome. CRD, cumulative reactive dose; DBPCFC, double blind placebo-controlled food challenge; ED, eliciting dose; EPIT, epicutaneous immunotherapy; FAIM, Food Allergy Independent Measure; FAQLQ, Food Allergy Quality of Life Questionnaire; OIT, oral immunotherapy.

### Desensitization

Desensitization is the most commonly reported efficacy outcome in FA trials [13^▪▪^] and is typically assessed using a double-blind, placebo-controlled food challenge (DBPCFC). However, the methods and reporting practices for DBPCFCs are highly variable, leading to significant heterogeneity and inconsistency. Some trials report the highest single tolerated dose, while others focus on the cumulative dose tolerated. Definitions of desensitization also vary, from ability to tolerate a maintenance phase dose to achieving a specific dose during a successful exit oral food challenge [14].

Evidence from two systematic reviews suggest OIT is effective for achieving desensitization across different food allergens. A meta-analysis of eight randomized controlled trials (RCTs) for peanut OIT reported a relative risk (RR) of 11.32 [95% confidence interval (CI): 5.93–21.60] and a NNT of 2; an RR of 4.67 (95% CI: 2.66–8.21) was reported for egg OIT based on five RCTs, while cow's milk OIT demonstrated RR of 13.98 (95% CI: 3.51–55.65) based on four RCTs. Another review resulted in similar findings, demonstrating OIT ability to desensitize individuals with peanut OIT (RR of 9.9 (95% CI: 4.5–21.4, high certainty)) for tolerating 300 mg of peanut protein and an RR of 16.6 (95% CI: 8.0–34.4, high certainty) for tolerating 1000 mg, both with an NNT of 2. Similarly, desensitization rates for cow's milk and egg allergies were also high, with RRs of 5.7 (95% CI: 1.9–16.7, moderate certainty) and 8.9 (95% CI: 4.4–18.0, moderate certainty), respectively, each with an NNT of 2.

While desensitization is widely used as an efficacy outcome in FA trials, real-world examples, such as the development and adoption of OIT for peanut allergy, highlight the complexities and challenges in translating trial success into practical, widespread clinical use. The United States Food and Drug Administration (FDA) approved Palforzia, an OIT product for peanut allergy, in 2020. However, in September 2023, Nestlé announced the divestment of Palforzia, its peanut allergy treatment business, to Stallergenes Greer [15]. This decision followed a strategic review initiated due to slower-than-expected adoption by patients and healthcare professionals. Several reasons may be responsible for this slow uptake, such as uncertainty about the clinical impact of the reported threshold changes after Palforzia treatment, logistical challenges of treatment protocols requiring medical supervision, patient and provider hesitancy due to concerns over long-term efficacy and side effects, high costs limiting accessibility, and the need for significant lifestyle adjustments. This meant it was difficult to evaluate and communicate the expected benefits and risks of the treatment, to make informed clinical management decisions.

Another treatment modality that demonstrated promising results with regards to increase in tolerance threshold is epicutaneous immunotherapy (EPIT), which involves allergen exposure through the skin. At present, EPIT is under investigation, with products like Viaskin Peanut undergoing clinical trials [16^▪▪^]. Despite these developments, achieving long-term SU posttreatment remains inconsistent, and concerns about safety, particularly the risk of adverse reactions during treatment, persist [17].

In February 2024, FDA also approved the biologic treatment omalizumab for reducing allergic reactions, including anaphylaxis, that may occur with accidental exposure to one or more foods in adults and children aged one year and older with FA. This approval was primarily based on data from the Phase III OUtMATCH trial [18^▪▪^], which demonstrated that omalizumab significantly increased the threshold amount of allergens such as peanuts, milk, egg, wheat, and tree nuts that individuals with FA could tolerate without experiencing allergic reactions. Real world evidence of the above-mentioned treatment modalities in routine care remains to be seen.

### Sustained unresponsiveness

SU, also referred to as ‘remission’, is broadly defined as the absence of clinical reactivity to a food allergen for a specific period after the cessation of allergen immunotherapy [6^▪^,^13^^▪▪^,^14^]. This definition suggests a lasting change in the immune system's response to an allergen, allowing individuals to safely consume food without allergic symptoms even after discontinuing treatment. The long-term effects of OIT were documented, with several studies indicating that at least a proportion of individuals maintain their ‘desensitized’ state for months or even years after discontinuing treatment, but the number of trials looking at long-term SU is still small [8^▪^]. Some studies have demonstrated that EPIT can also lead to SU, although the duration and reliability of this response may vary among individuals [19,20].

Recent evidence from two systematic reviews suggested the efficacy of OIT in achieving SU, though limitations remain. The first review [6^▪^] demonstrated a significant increase in SU rates with peanut OIT, reporting a RR of 7.74 (95% CI: 2.90–20.69) and a NNT of 4 to achieve SU 6–12 months after treatment cessation. Similar efficacy was observed for hen's egg OIT (RR 6.91; 95% CI: 1.67–28.57), although fewer studies measured SU for this allergen. The authors highlighted variability in trial designs, including differences in treatment duration, maintenance doses, and timing of follow-up food challenges, underscoring the need for standardized protocols. The second review [20] reported peanut OIT increasing the proportion of children achieving SU, with an RR of 8.8 (95% CI: 1.2–61.6, low certainty) and an NNT of 4. Hen's egg OIT resulted in similar outcomes, with an RR of 7.1 (95% CI: 1.7–29.4, low certainty) and an NNT of 4. While these findings confirm the potential of OIT to induce SU, the evidence remains less certain compared to desensitization outcomes.

Despite promising results associated with OIT and EPIT, challenges remain in achieving consistent and long-lasting SU across diverse patient populations. A multitude of different factors such as the duration of therapy, the specific allergen involved, and individual patient characteristics can influence the outcomes of immunotherapy [20,21]. While some patients may achieve long-term tolerance, others may experience a return of sensitivity after discontinuation of treatment [21]. Additionally, the risk of adverse reactions during immunotherapy, particularly with OIT, necessitates careful patient selection and monitoring throughout the treatment process [20,21].

While some trials may report successful desensitization, the lack of SU assessment, particularly in the absence of standardized definitions can obscure whether these patients truly achieve a state of immunity or temporary tolerance [22]. With exploration of other treatment modalities such as biologics, which has shown promise in enhancing allergen thresholds during accidental exposures, it becomes critical to establish consistent outcome measures that reflect both clinical efficacy and QoL improvements [8^▪^], and their relationship. While SU is intuitively a relevant outcome measure, its relationship to QOL is uncertain, and may depend on the advice and behaviours that accompany successful SU.

## DESIRED OUTCOME LANDSCAPE IN FOOD ALLERGY TRIALS

### Challenges in outcome selection for evidence synthesis

Systematic reviews assessing the efficacy of FA interventions face several challenges related to outcome selection and reporting (Table 2). Most substantial issues are similar to the ones observed in clinical trials and related to heterogeneity of outcome measures, including desensitization, SU, underreporting of adverse reactions, and most importantly, quality of life (QoL) [23^▪▪^]. Efficacy outcomes often lack standardized definitions and adverse events are inconsistently reported, with many studies focusing solely on severe reactions, limiting comprehensive safety assessments [17].

**Table 2:** Recent key systematic reviews on food allergy treatments

Author, year	Study designs included	Population	Intervention	Outcomes	Key findings (RR/OR (95%CI))	Number of studies in the analysis	Potential limitations
Lodge *et al.*, 2023 [6^▪^]	RCTs	Mixed population	OIT	DS peanut	11.32 (5.93–21.60)	8	Heterogeneous study designs and methods, lack of allergy confirmation in some studies, blending of ITT and PP analyses, limited generalizability to high-risk populations, underreporting of adverse events, short follow-up for SU, and high risk of allergic reactions.
				DS hen's egg	4.67 (2.66–8.21)	5	
				DS cow's milk	13.98 (3.51–55.65)	4	
				SU peanut	7.74 (2.90–20.69)	3	
				SU hen's egg	6.91 (1.67–28.57)	2	
				Adverse reactions peanut (adrenaline use)	2.96 (1.63–5.35)	8	
				Adverse reactions hen's egg (adrenaline use)	1.71 (0.42–6.92)	6	
				Adverse reactions cow's milk adrenaline use)	8.45 (2.02–35.27)	4	
				HRQL	One on peanut OIT reported improvements in HRQL, though unblinding before the second QoL assessment may have influenced results. Another more recent study showed improved QoL in the treatment group. A third study, which compared OIT to avoidance, also found increased QoL in those receiving OIT. However, other research on QoL outcomes has shown mixed results.	3	
Zuberbier *et al.*, 2023 [51]^a^	RCTs, CCTs, and observational	Mixed population	OMA vs. pre-OMA	Increase in tolerated dose (multiple foods)	24.88 (6.35–97.45)	4	High heterogeneity in treatment protocols and study designs. Small sample sizes and limited studies for key outcomes. High risk of bias and potential publication bias. Limited long-term efficacy and safety data. Industry sponsorship may introduce conflicts of interest.
			OMA+OIT vs. placebo	Increase in tolerated dose (multiple foods)	1.72 (1.16–2.55)	2	
			OMA+OIT vs. placebo	DS cow's milk	9.23 (2.70–31.60)	5	
			OMA+OIT vs. pre-OMA	Parental HRQoL	MD –0.76 (–1.40 to –0.12)	1	
			OMA+OIT vs. pre-OMA	Patient's HRQoL	MD –2.00 (–2.30 to –1.71)	1	
			OMA vs. pre-OMA	Parental judgment of HRQoL (multiple foods)	MD 26.91 (23.72–30.10)	1	
			OMA vs. pre-OMA	Patient's judgment of HRQoL (multiple foods)	MD 26.15 (20.03–32.28)	1	
Riggioni *et al.*, 2024 [23^▪▪^]	RCTs in phase II or higher and CCT	Mixed population	OIT	DS peanut	11.94 (1.76–80.84)	11	High heterogeneity in study designs, outcomes, and populations; inclusion of studies with unclear or high risk of bias; limited evidence on long-term efficacy and safety; underreporting of adverse events; lack of standardised DS protocols and outcome measures; and limited generalisability to broader populations.
				DS cow's milk	5.88 (2.27–15.18)	10	
				DS egg	3.43 (2.24–5.27)	17	
			SLIT	DS peanut	3.00 (1.04–8.66)	3	
			EPIT	DS peanut	2.16 (1.56–3.00)	3	
			Omalizumab	DS food allergy (different allergens)	2.17 (1.22–3.85)	8	
			OIT, SLIT, EPIT, Omalizumab	HRQoL (different allergens)	Current literature lacks head-to-head studies comparing the impact of treatments and studies on the optimal combination and duration of treatment.	NA	
Cao *et al.*, 2021 [24]	RCTs, CCTs, and observational	Mixed population	OIT (before-after)	HRQoL (different allergens)	Mean change: −1.25 (95% CI: −1.77 to −0.72); *P* < .001; I^2^ = 87%	7	High heterogeneity in study designs, HRQoL measures, and follow-up intervals; limited long-term data on sustained HRQoL benefits; lack of consistency in tools for measuring caregiver burden; and potential variability in results based on allergen type and baseline HRQoL.
		Children (ages 0–17 years)	OIT vs. placebo	HRQoL improvement	SMD: −0.56 (95% CI: −0.92 to −0.20); *P* = .007; I^2^ = 42%	5	
		Caregivers of children with FA	OIT	Parental burden (different allergens)	FAQLQ-PB mean change: −2.67 at 18 months, −2.09 at 6 months	1	
de Silva *et al.*, 2022 [20]	RCTs	Mixed population	OIT	DS peanut	9.9 (4.5–21.4)	7	High heterogeneity in study designs and populations, limited data on long-term outcomes, underreporting of adverse events, lack of standardised protocols for immunotherapy regimens.
			OIT	DS cow's milk	5.7 (1.9–16.7)	7	
			OIT	DS hen's egg	8.9 (4.4–18.0)	6	
			OIT	Adverse reactions cow's milk	3.9 (2.1–7.5)	6	
			OIT	Adverse reactions hen's egg	7.0 (2.4–19.8)	6	
			Immunotherapy	Adverse reactions peanut	1.1 (1.0–1.2)	7	
			Immunotherapy	HRQoL	There was little information about the impact on quality of life.	NA	
		Children	EPIT	DS peanut	2.6 (1.8–3.8)	3	
			OIT	SU peanut	8.8 (1.2–61.6)	1	
Tang *et al.*, 2022 [52]	RCTs for OIT and RCTs and trials and controlled comparisons for biologicals	Children (0–18 years)	OIT	DS cow's milk	7.35 (2.82–19.13)	11	High heterogeneity in study designs and protocols; inconsistent definitions of DS and SU; limited evidence for long-term outcomes and HRQoL improvements; underreporting of adverse events; and insufficient data to analyse immunological changes and adjuvant therapies comprehensively.
		Children >3 years		DS cow's milk	18.05 (6.48–50.26)	7 (subset of 234 patients)	
		Children (0–18 years)		Partial DS cow's milk	9.94 (2.8–34.37)	5	
		Children (0–18 years)		SU cow's milk	Salmivesi *et al.*[53] −23/28 and 22/28 patients tolerated cow's milk at 6–12 months and 3–3.5 years post-DS, while Maeda *et al.*[47] reported 7/8 patients tolerating >100 ml cow's milk 2 years poststudy.	2	
		Children (0–18 years)		SAEs	2.2 (0.59–8.22)	6	
		Children (0–18 years)		Non-SAEs	4.21 (2.9–6.13)	6	
		Children (0–18 years)		Epinephrine use during intervention	6.45 (1.53–27.11)	6	

CCTs, controlled clinical trials; DS, desensitization; EPIT, epicutaneous immunotherapy; HRQoL, health-related quality of life; ITT, intention-to-treat; OIT, oral immunotherapy; OMA, omalizumab; PP, per protocol analysis; RCTs, randomized-controlled trials; SAEs, serious adverse events; SU, sustained unresponsiveness.

a Zuberbier *et al.* (2023) reported OMA, either as monotherapy or combined with OMA+OIT, significantly improved QoL for patients with FA and their carers (data from two small sample size observational studies).

Another limitation is related to short follow-up durations in many studies, which do not allow for appropriate evaluation of long-term efficacy, particularly for SU [13^▪▪^]. This is a feature of many clinical trials in many disease areas, since long-term foll0w-up and retention of clinical trial populations is very challenging. Population heterogeneity, such as mixed-age groups and inconsistent allergy confirmation, can influence the generalizability of findings.

QoL as an outcome, and other patient-centred outcomes such as measures of caregiver burden, remain largely underexplored in clinical trials of FA treatment, with inconsistent measurement tools further complicating interpretation [23^▪▪^,^24^]. To address these challenges, the adoption of COS for FA is essential. A standardized measurement framework that incorporates patient-centred outcomes, together with agreed definitions of constructs, scales and timeframes, would allow for the comparison of efficacy of FA treatments between centres, trials, and/or settings [9].

### Core outcomes sets as a proposed solution to heterogeneity

COS have become increasingly important in clinical research and trials, addressing challenges related to outcome heterogeneity and reporting bias [10]. These standardized sets of outcomes, developed through consensus-based approaches, ensure the inclusion of diverse perspectives and improve the quality of RCTs and systematic reviews [25].

The development of COS typically involves mixed-methods approach to identify the most critical outcomes to be measured for a specific condition or intervention. Over the past two decades, many COS initiatives have been launched across medical fields, not just providing information about optimal outcomes to be assessed and ways of assessment but substantially impacting clinical trial conduct. Example of the successful adoption of COS has been reported by Kirkham *et al.*, who found 81% uptake of the rheumatoid arthritis COS in clinical trials, demonstrating its growing influence in standardizing outcome measurement on real-life research [26].

Several efforts were undertaken in the allergy field, for instance by the Harmonizing Outcome Measures for Eczema (HOME) [27], core outcome set for therapeutic studies in eosinophilic esophagitis (COREOS) [28], Core Outcome Measures sets for paediatric and adult Severe Asthma (COMSA) [29], and very recently COMFA initiatives [12^▪▪^] providing guidance for clinical trial conduct.

COMFA initiative aimed to develop COS specifically for IgE-mediated FA clinical trials and observational studies of interventions. The process included a comprehensive review to generate an extensive list of 39 potential outcomes for further consideration. These outcomes were refined through discussions among researchers, healthcare professionals, and individuals with lived experience of FA. This led to a more focused list of 13 outcomes, which were evaluated through a two-round, online-modified Delphi process involving 778 participants from 52 countries. Following the Delphi rounds, a hybrid consensus meeting was held, combining in-person and online participation, to finalize the COS.

The consensus process identified “allergic symptoms” and “QoL” as core outcomes that should be measured and reported. While other outcomes, such as “remission/SU” and “desensitization” were considered important, they did not meet the predefined thresholds for inclusion. The emphasis on allergic symptoms and QoL reflects the importance of addressing patient-centred outcomes in FA research. The project also underscored the need for future work to standardize tools for measuring these outcomes. It is not unusual to see symptoms and/or disease control and particularly QoL among critical outcomes selected as a result of a COS development process and they were similarly prioritized in the allergy field for eosinophilic esophagitis [28], eczema [27], asthma [29] as well as chronic conditions representing other medical fields [30–32]. This alignment with broader trends in COS development highlights the universal importance of “patient-centred” outcomes such as QoL and symptom reporting/control in driving meaningful advancements in clinical research.

### Quality of life patient-perspective

Many individuals face challenges in social environment where food plays a central role, such as dining out or attending different events [33,34], often leading to feelings of social isolation. These difficulties are particularly challenging and important for children and young people, who may experience frustration and exclusion as they navigate peer acceptance while managing their FA [3,35]. Stigma also remains a challenge as it may engender feelings of shame or embarrassment, particularly in social contexts, further isolating individuals and negatively impacting their mental health [3]. For carers, especially parents of children with FA, the condition is associated with an additional burden [3]. They frequently report high levels of stress and anxiety related to ensuring their child's safety, which can also impact family dynamics and reduce overall QoL. There are also developmental concerns. Although in the early years (when FA is typically diagnosed), the focus is mainly on the parent, experiences in early childhood play an active role in the development of brain and behaviour that may carry a long-lasting influence [36]. For young children who perceive the world as a threatening place, a wide range of conditions have been shown to trigger anxious behaviours that then impair ability to learn and to interact socially with others.

Research suggests that the primary burden of FA is its QoL impact rather than the direct effects of acute reactions. People with lived experience of FA therefore primarily seek improvements in QoL rather than a definitive ‘cure’ [3,7,37]. During the recent COMFA COS development process, everyone agreed that QoL is a critical outcome which must be measured in all clinical trials of FA treatment, as well as observational studies of FA interventions, while acknowledging that a permanent cure cannot be guaranteed [12^▪▪^]. However, the importance of providing a clear and balanced description of the “Remission/SU” outcome was also emphasized.

While achieving SU is emphasized as a priority for clinical research, and particularly efficacy assessment [7,12^▪▪^], many people are satisfied with ongoing desensitization that allows for improved day-to-day functioning without eliminating the need for vigilance. This suggests that a measurable reduction in dietary restrictions and associated QoL measures should be detectable, if the desensitization or SU has done something useful for patients. Despite the growing emphasis on improving QoL in the management of FA, it is still assessed and reported inconsistently across clinical trials. When QoL is assessed, conflicting results emerge, reflecting variability in study designs, patient populations, and measurement tools [23^▪▪^,^24^]. Some of the major clinical trials that have reported efficacy in desensitizing patients, failed to provide improvement in the QoL [16^▪▪^,^17^], and sometimes QoL has not been even reported [16^▪▪^].

The lack of measured improvement in QoL in some FA treatment trials may be due to a loss of variation across samples due to a ‘regression to the mean’. For example, participation in a trial may be a positive experience for caregivers, with a reduction in risk and uncertainty and an awareness of potential long-term benefits, but any improvement in QoL may be weakened by their children's experience of consuming disliked foods since they are preoccupied more with immediate symptoms and experiences. However, there may also be a lack of genuine QoL impact, and this may relate to relatively conservative advice regarding allergen avoidance and reaction risk which often accompanies FA treatments in trials. More holistic interventions aimed at QoL improvement as a primary outcome, and longer term assessments (beyond trial end) are needed as QoL improvement may take time to manifest, as confidence builds following successful desensitisation or remission [38^▪^,^39^,^40^].

### Allergic symptoms

Assessment of allergic symptoms in FA clinical trials typically incorporate both patient-reported as well as investigator-reported measures. Patient-reported assessments involve self-reported symptoms recorded by participants, often through daily diaries or surveys as a part of their daily life. These are designed to capture the frequency and severity of allergic reactions, including common allergic manifestations such as skin, gastrointestinal and respiratory signs and symptoms [18^▪▪^]. Investigator-reported assessments, on the other hand, are conducted by healthcare professionals and usually include DBPCFC to confirm the presence of an allergy and quantify the degree of tolerance increase to specific allergen [13^▪▪^]. The timing of symptom assessments is also critical. Some trials evaluate symptoms at multiple time points, including baseline, during treatment, and at follow-up, to track changes in allergic reactions over time and this longitudinal approach allows more explicit evaluation of intervention effect on trial participant, in reducing the frequency and severity of allergic symptoms.

The reduction of ‘allergic symptoms’ i.e. risk associated with accidental allergen exposure was also prioritized in COMFA as a critical COS for FA intervention studies [37]. For instance, individuals undergoing OIT report valuing a “buffer” against unintentional exposure. This buffer may manifest decreased reaction severity, increased time to react, or higher exposure thresholds. Such outcomes in turn can have a positive impact on QoL, by increasing confidence in engaging with daily activities, such as dining out or traveling, even if treatment does not entirely eliminate preexisting anxiety or avoidance behaviours [37,41]. It is likely that in this context, where allergic symptoms are changed in nature, frequency or timing, the advice that accompanies OIT may be an important moderator of any QoL impact associated with changes in allergic symptoms.

Assessment of symptoms in context of clinical trials, incorporated as a part of an efficacy evaluation, is normally related to exit OFC [14]. Another important aspect is related to using different criteria to define the tolerated dose during OFCs, which was shown to lead to variability in results, with discordance reported in one in five individuals. This is related to whether less objective symptoms, such as mild or subjective reactions are included, and can impact the interpretation of tolerated doses [42^▪^]. The use and evaluation of patient-reported outcome measures, whether they reflect upon QoL and/or symptoms, in clinical trials may enhance the potential for uptake, effectiveness and sustainability in real world settings [43]. They can act as one of the tools to assess whether the results from trials will translate well to the target population [40].

## SUMMARY

Prioritization of standardized and patient-centred outcome measures in FA research is critically important. The variability in outcome selection and reporting in clinical trials limits comparability of findings and the synthesis of evidence, particularly in the context of emerging interventions such as OIT/EPIT. QoL has often been omitted from clinical trial reports in the field, even when recorded. Development of a COS, such as done by the COMFA initiative, represents a step forward in addressing these challenges. By identifying “allergic symptoms” and “QoL” as the most critical outcomes, COMFA has reiterated the necessity of aligning clinical research with patient priorities and lived experiences. The use and evaluation of PROMs in clinical trials may enhance the potential for uptake, effectiveness and sustainability in real world settings. Although outcomes were defined, future efforts should focus on standardizing measurement tools, enhancing long-term outcome assessment, and addressing gaps in the reporting of secondary outcomes, such as caregiver burden and health-related QoL.

## Acknowledgements

*During the preparation of this work the authors used Artificial Intelligence platforms (Writefull AI, Scispace and ChatGPT) in order to improve the grammatical structure and readability. After using this tool, the authors reviewed and edited the content as needed and take full responsibility for the content of the publication.*

### Financial support and sponsorship

*None.*

### Conflicts of interest

*There are no conflicts of interest.*

## REFERENCES AND RECOMMENDED READING

Papers of particular interest, published within the annual period of review, have been highlighted as:

▪ of special interest
▪▪ of outstanding interest
